# New Chondrosarcoma Cell Lines with Preserved Stem Cell Properties to Study the Genomic Drift During In Vitro/In Vivo Growth

**DOI:** 10.3390/jcm8040455

**Published:** 2019-04-04

**Authors:** Veronica Rey, Sofia T. Menendez, Oscar Estupiñan, Aida Rodriguez, Laura Santos, Juan Tornin, Lucia Martinez-Cruzado, David Castillo, Gonzalo R. Ordoñez, Serafin Costilla, Carlos Alvarez-Fernandez, Aurora Astudillo, Alejandro Braña, Rene Rodriguez

**Affiliations:** 1 University Central Hospital of Asturias—Health and Research Institute of Asturias (ISPA), 33011 Oviedo, Spain; reyvazquezvero@gmail.com (V.R.); sofiatirados@gmail.com (S.T.M.); o_r_e_s_@hotmail.com (O.E.); aidarp.finba@gmail.com (A.R.); laurasd.finba@gmail.com (L.S.); juantornin@gmail.com (J.T.); lucialmc24@gmail.com (L.M.-C.); 2 University Institute of Oncology of Asturias, 33011 Oviedo, Spain; 3 CIBER in Oncology (CIBERONC), 28029 Madrid, Spain; 4 Disease Research and Medicine (DREAMgenics) S.L., 33011 Oviedo, Spain; david.castillo@dreamgenics.com (D.C.); gonzalo.ordonez@dreamgenics.com (G.R.O.); 5 Department of Radiology of the Servicio de Radiología of the University Central Hospital of Asturias, 33011 Oviedo, Spain; costillaserafin@uniovi.es; 6 Department of Medical Oncology of the Servicio de Radiología of the University Central Hospital of Asturias, 33011 Oviedo, Spain; carlos.alvfer@gmail.com; 7 Department of Pathology of the Servicio de Radiología of the University Central Hospital of Asturias, 33011 Oviedo, Spain; astudillo@hca.es; 8 Department of Traumatology of the University Central Hospital of Asturias, 33011 Oviedo, Spain; albravigil@gmail.com

**Keywords:** chondrosarcoma, primary cell lines, cancer stem cells, whole exome sequencing, genomic drift, animal model, cancer preclinical model

## Abstract

For the cancer genomics era, there is a need for clinically annotated close-to-patient cell lines suitable to investigate altered pathways and serve as high-throughput drug-screening platforms. This is particularly important for drug-resistant tumors like chondrosarcoma which has few models available. Here we established and characterized new cell lines derived from two secondary (CDS06 and CDS11) and one dedifferentiated (CDS-17) chondrosarcomas as well as another line derived from a CDS-17-generated xenograft (T-CDS17). These lines displayed cancer stem cell-related and invasive features and were able to initiate subcutaneous and/or orthotopic animal models. Different mutations in Isocitrate Dehydrogenase-1 (*IDH1*), Isocitrate Dehydrogenase-2 (*IDH2*), and Tumor Supressor P53 (*TP53*) and deletion of Cyclin Dependent Kinase Inhibitor 2A (*CDKN2A*) were detected both in cell lines and tumor samples. In addition, other mutations in *TP53* and the amplification of Mouse Double Minute 2 homolog (*MDM2*) arose during cell culture in CDS17 cells. Whole exome sequencing analysis of CDS17, T-CDS17, and matched patient samples confirmed that cell lines kept the most relevant mutations of the tumor, uncovered new mutations and revealed structural variants that emerged during in vitro/in vivo growth. Altogether, this work expanded the panel of clinically and genetically-annotated chondrosarcoma lines amenable for in vivo studies and cancer stem cell (CSC) characterization. Moreover, it provided clues of the genetic drift of chondrosarcoma cells during the adaptation to grow conditions.

## 1. Introduction

Chondrosarcoma is a malignant cartilage-forming tumor that represents, with approximately 25% of the cases, the second most common bone sarcoma. The most common subtype, representing 80% of the total, is the primary or conventional central chondrosarcoma which is characterized by the pathological formation of hyaline cartilage within the medullar cavity [1]. Other less common subtypes include the periosteal chondrosarcoma, which occurs in the surface of the bone, and secondary chondrosarcoma, which arises in benign lesions such as enchondroma, frequently associated to Ollier disease or Maffucci syndrome, or osteochondroma [1]. The dedifferentiated chondrosarcoma is a distinct variety of chondrosarcoma, accounting for approximately a 10% of the cases. This subtype is characterized by the presence of a low-grade well-differentiated cartilaginous tumor juxtaposed to a high-grade anaplastic sarcoma [1].

Within the complex cytogenetic scenario characteristic of chondrosarcomas, a few chromosomal alterations and gene mutations are frequently found. Thus, mutations in Isocitrate Dehydrogenase-1 *(IDH1*) and -2 (*IDH2*) are found in 87% of enchondromas, up to 70% of conventional central chondrosarcomas and 54% of dedifferentiated chondrosarcomas and may drive sarcomagenic processes [2,3,4]. In addition, mutations in the major cartilage collagen (*COL2A1*) or Tumor Supressor P53 (*TP53*) genes have been found in approximately 35% and 20% of the chondrosarcomas respectively [5,6]. Other frequent copy number variations, such as the amplification of the 12q13 region, containing Mouse Double Minute 2 homolog (*MDM2*) and the cyclin-dependent kinase 4 genes, and the deletion of the 9p21 region, which includes the Cyclin Dependent Kinase Inhibitor 2A (*CDKN2A*) locus, contribute to the deregulation of the p53 and Retinoblastoma (RB) pathways [5,7].

Wide surgical resection is the mainstay therapeutic option for localized chondrosarcomas. However, these tumors often show high local recurrence and metastatic potential. Furthermore, chondrosarcomas are inherently resistant to conventional chemo and radiotherapy and nowadays there are no effective treatments available for metastatic or inoperable tumors [8,9,10,11,12]. Proposed mechanisms of chemoresistance include the role of the cartilaginous extracellular matrix as a barrier for drug diffusion, the overexpression of members of the adenosine triphosphate (ATP) binding cassette transmembrane family of efflux pumps and antiapoptotic proteins, and the presence of a high percentage of low proliferating/quiescent cells [9,11,12]. Interestingly, some of these features are related to those described for tumor cell subsets presenting stem cell properties (cancer stem cells, CSC). These CSC subpopulations have been characterized in several subtypes of sarcomas and associated to the expression/activity of pluripotency factors, like Sex Determining Region Y-Box 2 (SOX2), stem cell markers, like Aldehyde Dehydrogenase 1 Family Member A1 (ALDH1), or to the ability to grow as floating clonal spheres (tumorspheres) [13,14]. CSC subpopulations have been barely characterized in chondrosarcoma and therefore new models amenable for the study of these subpopulations are needed to find vulnerabilities against these drug-resistant subpopulations.

Despite their failure to completely reproduce the genetic and microenvironmental conditions of tumors, cell lines are still indispensable models to investigate altered mechanisms in cancer, to study CSC subpopulations, and to serve as drug-screening platforms in a high-throughput and logistically simple and rapid way [15,16,17]. To our knowledge, 14 tumor-derived chondrosarcoma lines, corresponding to seven conventional, five dedifferentiated, and two secondary chondrosarcomas, have been reported so far [18,19,20,21,22,23,24,25,26,27]. In this work, we succeeded in establishing three cell lines from two secondary (CDS06 and CDS11) and one dedifferentiated (CDS-17) chondrosarcoma as well as another cell line derived from a CDS-17 xenograft (T-CDS17). We studied their tumorigenic and invasive potential, characterized the present CSC subpopulations, and analyzed the most relevant chondrosarcoma-related genetic alterations both in the cell lines and their original tumors. Furthermore, using a whole exome sequencing (WES) approach in CDS17 and T-CDS17 cells and their matched patient samples we were able to track the genomic adaptation of tumor cells to in vitro and in vivo growth.

## 2. Experimental Section

### 2.1. Establishment of Cell Lines

Human samples and data from donors included in this study were provided by the Principado de Asturias BioBank (PT17/0015/0023) integrated in the Spanish National Biobanks Network upon obtaining of written informed consent from patients. All experimental protocols have been performed in accordance with institutional review board guidelines and were approved by the Institutional Ethics Committee of the University Central Hospital of Asturias (approval number: 45/16). This study was performed in accordance with the Declaration of Helsinki.

Tumor samples were subjected to mechanical disaggregation followed by an enzymatic dissociation using MACS^®^ Tissue Dissociation Kit and the GentleMACS Dissociator system (Miltenyi Biotec, Bergisch Gladbach, Germany). At the end of the incubation, culture medium (DMEM-Dulbecco’s Modified Eagle Medium supplemented with 10% FBS, 2 mM L-glutamine, 100 U/mL penicillin and 100 µg/mL streptomicin) was added and the cell suspension was filtered to remove clusters. Tumor cells were collected by centrifugation, resuspended in fresh culture medium and seeded in culture flasks. As an alternative protocol to derived cell lines, some fresh tumor specimens were cut into several small fragments, transferred to dry 25 cm^2^ culture flasks, covered with a drop of medium and incubated until outgrowth of tumor cells was observed. Cell cultures derived by both methods were subcultured when they reached 80–90% confluence. As a procedure to select tumoral cells and rid of stromal cells, we performed soft agar colony formation assays using the CytoSelect^TM^ 96-Well Cell Transformation Assay Kit (Cell Biolabs Inc, San Francisco, CA, USA). Cells able to form colonies under these anchorage-independent growth conditions are supposed to be transformed. These colonies were recovered, left to attach to plastic substrate and grow in culture medium as normal adherent cultures. Short Tandem Repeat (STR) analyses were performed to compare the identity of cell lines with matched tumor sample (Supplemental Information).

### 2.2. Tumorsphere Culture

The tumorsphere formation protocol was previously described [28].

### 2.3. Western Blotting

Whole cell protein extraction and western blotting analysis were performed as previously described [29]. Antibodies used are described in Supplementary Materials.

### 2.4. Aldefluor Assay and Cell Sorting

Cells with high Aldehyde Dehydrogenase (ALDH) activity was detected and isolated using the Aldefluor^TM^ reagent (Stem Cells Technologies, Grenoble, France) as previously described [13].

### 2.5. Three-Dimensional Spheroid Invasion Assay

Invasion assays using 3D spheroids in the presence or not of dasatinib or PF-573228 (Selleckchem, Houston, TX, USA) were performed as previously described [30] (see Supplementary Materials information for details).

### 2.6. In Vivo Tumor Growth

All animal research protocols were carried out in accordance with the institutional guidelines of the University of Oviedo and were approved by the Animal Research Ethical Committee of the University of Oviedo prior to the study (approval code: PROAE 11/2014; date of approval: 9 December 2014). Experiments were done using female NOD.CB17/Prkdcscid/scid/Rj inbreed mice (Janvier Labs, St. Berthevin, France). For subcutaneous (s.c.) inoculations in the flanks of the mice, 5 × 10^5^ cells mixed 1:1 with BD Matrigel Matrix High Concentration (Becton Dickinson-BD Biosciences, Erembodegem, Belgium) previously diluted 1:1 in culture medium were injected. Tumor volume was determined using a caliper as previously described [31]. One month after inoculation, mice were sacrificed by CO_2_ asphyxiation and tumors were extracted and processed for histological analysis. Limited dilution assays (LDA) and calculation of tumor-initiating frequencies (TIF) was performed as described in Supplemental Information. For the intra-bone (i.b.) inoculation, mice were anesthetized with isoflurane and the leg was bent 90° in order to drill the tip of the tibia with a 25G needle before cell inoculation (2 × 10^5^ cells in 5 µl of culture media per mouse) using a 27G needle [32]. In these experiments, tumor growth was evaluated in the preclinical image laboratory of the University of Oviedo using a computed tomography (CT) system (Argus CT, Sedecal, Madrid, Spain) at week 8 after cell inoculation and a µCT system (SkyScan 1174, Bruker, Antwerp, Belgium) following mice sacrifice at week 12 (see Supplementary Materials for analysis conditions).

### 2.7. Histological Analysis

Human and xenograft samples were fixed in formol, decalcified using solutions with nitric acid (10%) or formic acid (4%)/chlorhydric acid (4%) and embedded in paraffin. Sections of 4-µm were stained with hematoxylin and eosin (H&E) as previously described [29].

### 2.8. MDM2 and CDKN2A Gene Copy Number Analysis

Genomic DNA was extracted using the QIAmp DNA Mini Kit (Qiagen, Hilden, Germany) and gene amplification was evaluated by real-time PCR. Reactions were carried out using the following primers: for MDM2 gene, Fw 5′-TGGCTGTGTTCAAGTGGTTC-3′ and Rv 5′-GTGGTGACAGGGTGCTCTAAC-3′; for CDKN2A gene, Fw 5′-CACATTCATGTGGGCATTTC-3′ and Rv 5′-TGCTTGTCATGAAGTCGACAG-3′ (Exon 3, recognizing both p14^ARF^ and p16^INK4^ sequences); and for the reference gene RPPH1 (ribonuclease P RNA component H1), Fw 5′-GAGGGAAGCTCATCAGTGG-3′ and Rv 5′-ACATGGGAGTGGAGTGACAG-3′. Dissociation curve analysis of all PCR products showed a single sharp peak and the relative gene copy number was calculated using the 2^−ΔΔCT^ method. For each set of samples, DNA from the corresponding healthy tissue was used as a calibrator.

### 2.9. Mutational Analysis of TP53, IDH1, IDH2, PI3KCA

Genomic DNA were amplified by PCR (Taq PCR Master Mix (2x), EURx Ltd. (Gdańsk, Poland)). The fragments analyzed include: exon 4 of *IDH1* and *IDH2* genes, identified as mutation hot spots in chondrosarcoma; exon 20 of the *PI3KCA* gene; and exons 4 and 6 of the *TP53* gene. Reactions were carried out using the forward and reverse primers detailed in Supplemental Information and the different PCR products were detected by gel electrophoresis in 1.5% agarose, showing a single band. Samples were purified and sequenced by Macrogen Ltd. (Madrid, Spain) and were aligned with the reference sequences of the genes using SnapGene^®^ 4.2.11 (GSL Biotech; available at snapgene.com).

### 2.10. Library Construction and WES

WES was performed by Macrogen (Seoul, Korea) using 1 µg of genomic DNA from each sample. DNAs were sheared with a Covaris S2 instrument and used for the construction of a paired-end sequencing library as described in the paired-end sequencing sample preparation protocol provided by Illumina. Enrichment of exonic sequences was then performed for each library using the Sure Select All Exon V6 kits following the manufacturer’s instructions (Agilent Technologies, Santa Clara, CA, USA). Exon-enriched DNA was pulled down by magnetic beads coated with streptavidin (Invitrogen, Carlsbad, CA, USA), followed by washing, elution, and additional cycles of amplification of the captured library. Enriched libraries were sequenced (2 × 101 bp) in an Illumina HiSeq4000 sequencer. WES results were processed using the bioinformatics software HD Genome One (DREAMgenics, Oviedo, Spain), certified with IVD/CE-marking (see Supplementary Materials for a comprehensive description of the exome analysis, [33,34,35,36,37,38,39,40,41,42,43,44,45,46,47]). The datasets generated during the study are available in the European Nucleotide Archive repository [48].

## 3. Results

### 3.1. Establishment of Patient-Derived Chondrosarcoma Cell Lines and Analysis of In Vivo Tumorigenic Potential

Surgically resected tumor samples from 11 patients diagnosed of chondrosarcoma at the Hospital Universitario Central de Asturias (Spain) were processed to establish primary cultures. Cultures from two secondary chondrosarcoma, CDS06 (associated with a previous osteochondroma) and CDS11 (presenting Ollier disease), and one from a dedifferentiated chondrosarcoma (CDS-17), were able to growth long term in vitro (Table S1 show an overview of patient and tumor characteristics). These cell lines were able to form colonies in soft agar, an in vitro transformation assay to test the ability of the cells to grow in anchorage independent conditions (Table 1). In order to select the more tumorigenic populations within the cultures, the colonies able to grow in soft agar were recovered and placed back in adherent culture to continue with the corresponding cell line development. Recovered cell lines could be passaged at least 20 times (Table 1) and their identity with the original tumor was confirmed by STR genotyping (Table S2).

**Figure 1:**
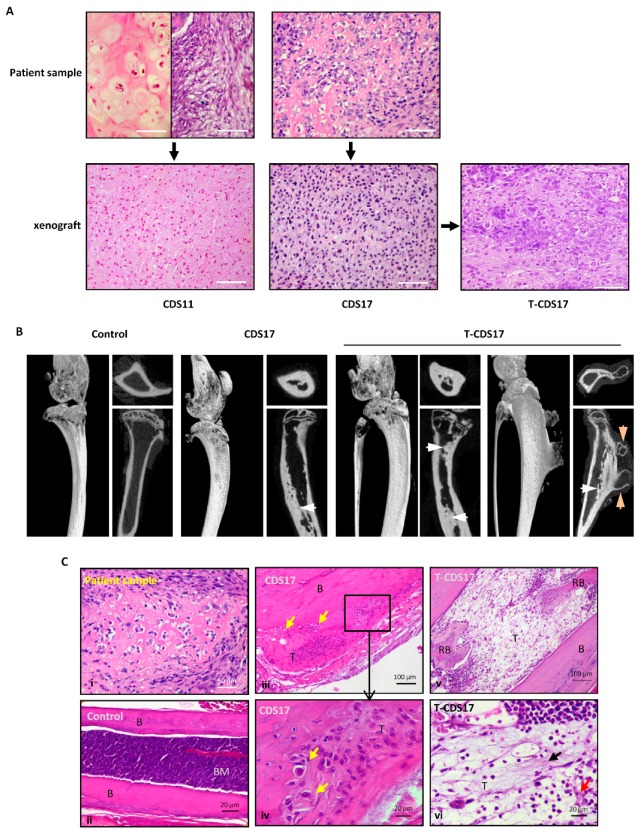
In vivo tumorigenicty of chondrosarcoma cell lines. (**A**) Histological analysis (H&E staining) of original patient tumors and tumors developed 1 month after subcutaneous (s.c.) inoculation of CDS11, CDS17, and T-CDS17 cell lines in immunodeficient mice. Two different areas of the CDS11 patient tumor sample are shown. scale bars = 150 µm. (**B**) Radiologic examination (µCT scan) of tumors developed after intra-bone (i.b.) inoculation of CDS17 and T-CDS17 cells in immunodeficient mice. Coronal, sagittal and axial images are shown. Compared to a control leg, intra-medullar formation of tumor bone/osteoid formation (white arrows) is shown in mice inoculated with both cell lines. In addition, a mouse inoculated with T-CDS17 cells presented an extra-medullar lesion compatible with the radiographic features of osteochondrosarcoma (orange arrows). (**C**) H&E staining of an original patient sample (i), a control leg (ii), and tumors formed after i.b. inoculation of CDS17 (iii and iv) and T-CDS17 (v and vi) cells lines. Chondroid.like cells (yellow arrows) and areas of fibrillar (black arrow) and amorphous (orange arrow) osteo-chondroid matrix are indicated. (B: bone; RB: reactive bone).

Two of the cell lines (CDS11, CDS17) were assayed for their ability to initiate tumor growth in vivo. Both of them were able to form small slow-growing tumors after subcutaneous (s.c.) inoculation in immunodeficient mice after 1 month (Figure 1A and Table 1). Following this, we generated a new cell line derived from a CDS17-xenograft tumor. Subsequent transplantation of this new cell line, T-CDS17, resulted in a more aggressive tumor growth (formation of significantly bigger tumors in similar latency periods), thus indicating that the tumor could be effectively propagated in vivo (Table 1). Histological analysis showed that the original CDS11 tumor was a malignant chondrosarcoma invading intra-trabecular bone matrix and presenting well differentiated and dedifferentiated areas. There was no inflammatory infiltrate and the dedifferentiated subcomponent displayed a mitotic index of 15 mitoses per 10 high power fields (HPF, 40X). The histology of tumors grown from the CDS11 line resembled that of the more undifferentiated/dedifferentiated areas of the original patient.

Tumor, with tumor cells distributed diffusely in a mesenchymal matrix. No well-differentiated component was found in these tumors. Inflammation was also absent and tumors showed 9 mitoses per 10 HPF (Figure 1A). The CDS17 patient sample was a high-grade chondrosarcoma displaying the characteristic chondroid differentiation, with tumor cells presenting pericellular matrix and surrounded by a chondroid extracellular matrix. There was no inflammation present in this tumor and its mitotic index was 18 mitoses per 10 HPF. Tumors derived from CDS17 and T-CDS17 cells lines maintained chondroid differentiation, with tumor cells presenting pericellular halos and embedded in a chondroid basophilic extracellular matrix. There were no inflammatory infiltrates in these tumors and its mitotic index was 17 and 25 mitoses per 10 HPF for CDS17 and T-CDS17 respectively (Figure 1A).

In an attempt to create more faithful animal models CDS17 and T-CDS17 cells were inoculated intra-tibia in immunodeficient mice. Both CDS17 (1 out of 2 mice) and T-CDS17 (2 out of 2 mice) cells were able to generate tumor growth in this orthotopic location. Computerized tomography (CT) analysis at day 50 after inoculation (Figure S1) and microCT analysis at the end point of the experiment (day 80) (Figure 1B) revealed the formation of tumors resembling the radiological features of human chondrosarcomas. These tumors showed the presence of a dotted pattern chondroid-like matrix inside the bone marrow cavity. In addition, one of the T-CDS17 generated tumors also displayed extra-medullar tumor growth, forming an osteochondroid exostosis-like lesion (Figure 1B and Figure S1). Histological sections of these orthotopically grown tumors also resembled the main features of the patient sample (Figure 1C(i)), with chondrogenic tumor cells presenting pericellular halos and embedded in cartilaginous matrix (Figure 1C(iii,iv)). In addition, legs inoculated with tumor cells showed bone marrow cavities filled by dedifferentiated mesenchymal cells producing fibrillar and amorphous osteo-chondroid matrix and presenting extensive areas of reactive bone (Figure 1C(v,vi)). None of tumors presented inflammatory component and they showed mitotic indexes between 7 (CDS17) and 10 (T-CDS17) mitoses per 10 HP.

### 3.2. Genetic Characterization of Chondrosarcoma Cell Lines

**Figure 2:**
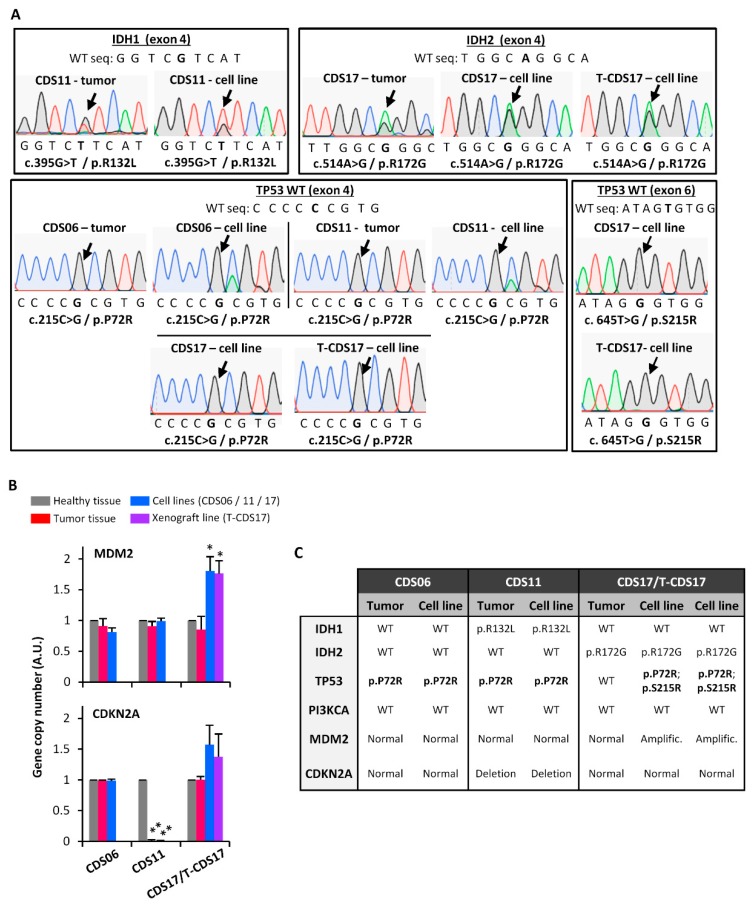
Genetic characterization of chondrosarcoma cell lines. (**A**) Sanger sequencing chromatograms showing mutations (black arrows) in *IDH1*, *IDH2*, and *TP53* genes present in the indicated tumors and cell lines. Reference wild type (WT) sequences are shown. (**B**) Gene copy numbers of the indicated genes were estimated by quantitative PCR on genomic DNA. Results are expressed relative to the corresponding healthy tissue sample and are the mean and standard deviation of three experiments (*: *p* < 0.05; ** *p* < 0.005; two-sided Student’s *t*-test). (**C**) Summary of the genetic characterization of the indicated chondrosarcoma cell lines and tumor samples. Homozygous mutations are highlighted in bold.

Sequencing analysis of common mutations in chondrosarcoma identified point mutations in *IDH1* (p.R132L) in CDS01 cells and *IDH2* (p.R172G) in CDS17 and T-CDS17 cells which were also detected in the corresponding patient tumor samples. Otherwise, CDS06 cells did not show any *IDH1* or *IDH2* mutations. Analysis of *TP53* (exons 4 and 6) revealed the presence of nonsynonymous homozygous in all cell lines. The single nucleotide variants (SNV) p.P72R was found in all cell lines and also in CDS06 and CDS11 patient samples, meanwhile the mutation p.S215R was only found in CDS17 and T-CDS17 cell lines but not in the corresponding patient sample (Figure 2A,C). Additional analysis of hot spot mutations in Phosphatidylinositol-4,5-Bisphosphate 3-Kinase Catalytic Subunit Alpha (*PI3KCA*) showed no alterations in any of the cell lines. Finally, copy number analysis showed a significant gain of *MDM2* in CDS17 and T-CDS17 cells which was not detected in the tumor sample and a homozygous deletion of *CDKN2A* (exon 3) in CDS11 cells and matched patient sample (Figure 2B,C).

### 3.3. WES of Chondrosarcoma Cell Lines and Clonal Evolution after In Vitro and In Vivo Growth

**Figure 3:**
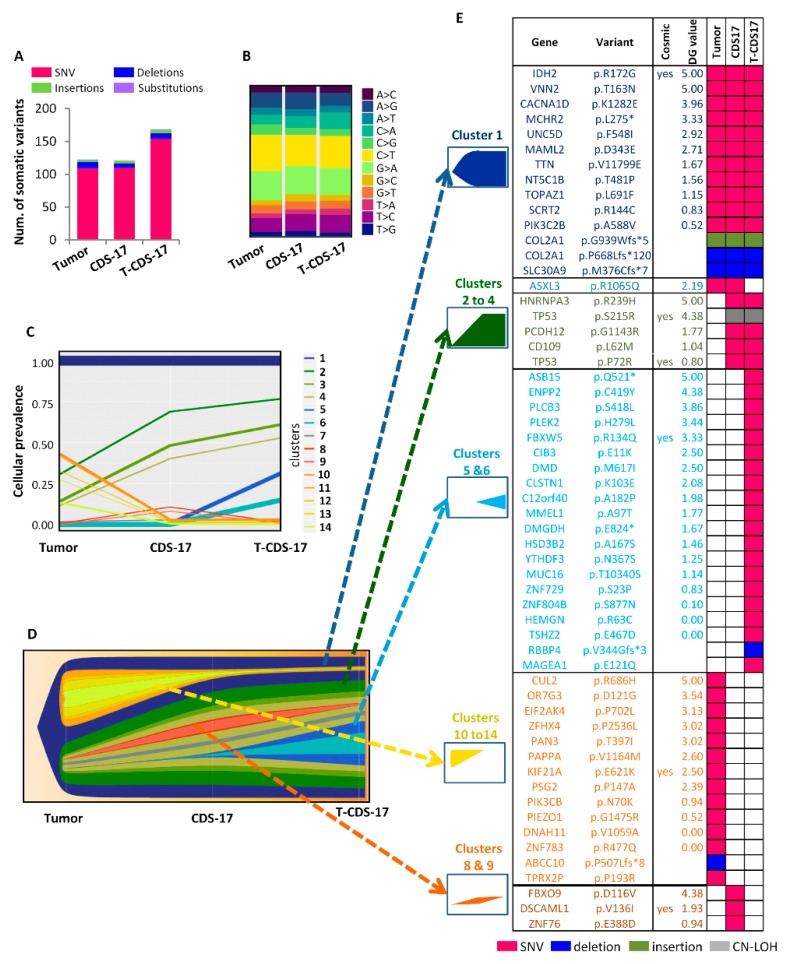
Clonal evolution of somatic mutations after in vitro and in vivo growth of chondrosarcoma cell lines. (**A**,**B**) Mutational burden data of a tumor patient sample and its derived cell lines CDS-17 and T-CDS17 obtained by whole exome sequencing (WES). The number and type of somatic mutations (**A**) and the profile of SNV transitions and transvertions (**B**) in each sample are shown. (**C**,**D**) Subclonal reconstruction was performed with PhyloWGS using WES data. The mean cellular prevalence estimates of mutation clusters in originating patient tumor sample and subsequent CDS17 and T-CDS17 cell lines are shown (**C**). Line widths represent the relative abundance of single nucleotide variants (SNVs) in each mutation cluster. Fish-plot representation of the different clusters in each sample is also shown (**D**). (**E**) List of non-synonymous somatic mutations detected in each cluster. Catalogue of Somatic Mutations in Cancer (COSMIC) status, DREAMgenics (DG) algorithm value, and mutation type are indicated.

To better characterize the genomic alterations present in these cells lines, we performed WES in the CDS17 line (passage 14), its xenograft-derived line T-CDS17 (passage 5) and their matched normal (non-tumoral) and tumor patient samples. The average nucleotide coverage in WES studies was approximately 115X, being selected for further analyses only the variants presenting more than 15 reads. Data from WES analysis of tumor the sample were compared to that of normal tissue DNA to exclude germline alterations. Tumor and CDS17 samples showed similar number of somatic mutations (123 and 121 respectively) whereas the T-CDS17 cell line displayed a slightly higher number of mutations (169), corresponding most of them to SNV in the three samples (Figure 3A). All samples displayed a similar profile of SNV transitions and transvertions (Figure 3B and Figure S2A). Similar to other type of tumors, C > T and G > A transitions were the most common mutations found in all samples (Figure 3B). To analyze the genomic evolution of tumor cells after in vitro/in vivo growth adaptation, we used variant allele frequency data of tumors, CDS17 and T-CDS17 samples to delineate the different clonal populations in each sample using the PhyloWGS, and FishPlot software. This analysis retrieves 14 clusters which evolved among samples (Figure 3C,D). The tumor sample contains nine clusters presenting cellular prevalence values higher than 0.05. Among them, cluster 1 is the one including a higher number of SNVs and must be the founder clone since the set of mutations that contain is present in virtually all the tumors cells (cellular prevalence equal to 1) and the other clones seem to derive from it. Notably, the cellular prevalence of cluster 1 is maintained in CDS17 and T-CDS17 samples, thus suggesting that most variants, including driver mutations, are kept by in the cell lines. In addition, cluster 7 remained unchanged in a small proportion of cells in all samples. On the other hand, Clusters 2–4 were positively selected while Clusters 10–14 almost disappeared during the adaptation to in vitro culture, as seen by their variation in cellular prevalence in the CDS17 line. Moreover, Clusters 5 and 6 emerged in the cell line T-CDS17 with a cellular prevalence of 0.25 and 0.15 respectively and were likely acquired during the in vivo growth of tumor cells in immunodeficient mice (Figure 3C,D).

**Figure 4:**
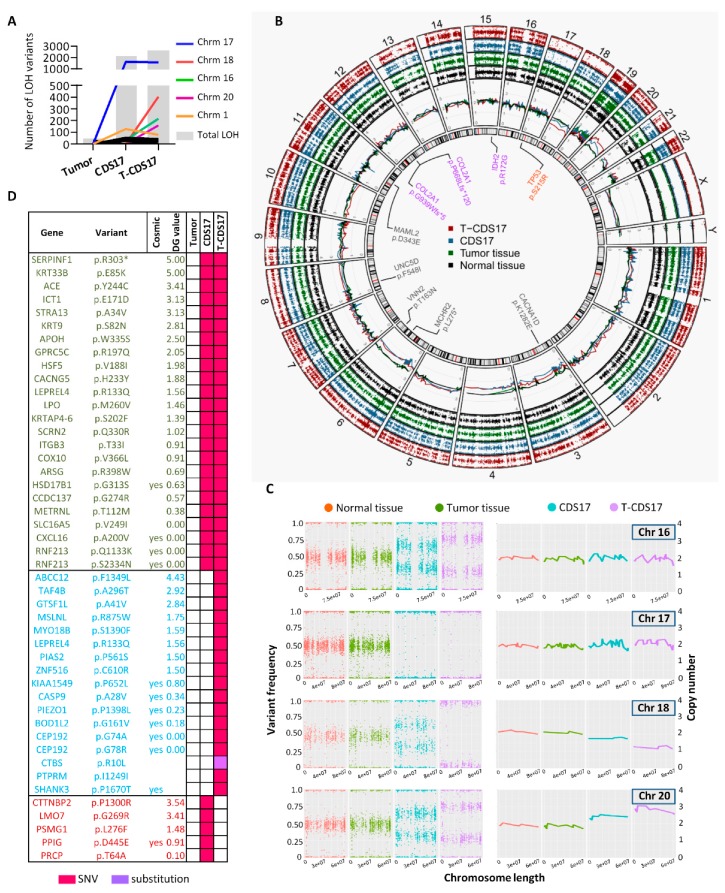
Genomic structural alterations after in vitro and in vivo growth of chondrosarcoma cell lines. (**A**) Number of LOH (total number and distribution between chromosomes) calculated from WES data of tumor, CDS17, and T-CDS17 samples. (**B**) Circular representation of the four samples sequenced through WES. Circles display from the inside outwards: (ring 1) chromosome ideogram, highlighting relevant previously reported (purple) or unreported (grey) somatic variants shared by tumor tissue, CDS17 and T-CDS17, as well as a TP53 homozygous mutation shared by CDS17 and T-CDS17 but not tumor tissue (orange); (ring 2) chromosome copy number (CN) of each sample based on normalized read counts; (rings 3–6) variant frequencies of common polymorphic positions (minimum allele frequency ≥0.01 in at least one major dbSNP population) in each sample. (**C**) Analysis of variant frequencies (left panels) and CN (right panels) extracted from (**B**) in the indicated chromosomes. Similar representations for other chromosomes are show in Figure S3B. (**D**) List of T-CDS17 cells. Conversely, a copy of chromosome 20 was gained in subset of CDS17 cells and in the entire population of T-CDS17 cells (Figure 4B,C). Finally, other copy number variations (CNVs) and/or shifts in variant frequencies affecting subpopulations of CDS-17 and/or T-CDS17 cells, was also detected in other chromosomes like 1, 2, 4, 5, 6, or 7 (Figure 4B and Figure S2B).

To select the most relevant somatic mutations included in each group of clusters presenting similar trends we compared tumor versus normal, CDS17 versus tumor and T-CDS17 versus CDS17 samples and filter the results to select variants with non-synonymous effect on coded proteins which presented variant allele frequencies >0.035 in tumor, CDS17 or T-CDS17 samples and maximum allele frequencies <0.01 in population databases (dbSNP, ExAC, ESP, and 1000 Genomes) (Figure 3E). Using this approach, we found a group of 14 mutations included in cluster 1 which were mutated in all the samples. Among these mutations, only the c.514A > G transition (p.R172G) in *IDH2*, previously detected by Sanger sequencing (Figure 2A), was listed in the Catalogue of Somatic Mutations in Cancer (COSMIC). In addition, we also selected two different mutations in the *COL2A1* gene previously found in chondrosarcoma [5,6]. Mutations in the rest of genes were not previously described in cancer. Notably, some of these unreported variants, including SNVs in Vanin2 (*VNN2*), Calcium Voltage-Gated Channel Subunit Alpha1 D (*CACNA1D*), Melanin Concentrating Hormone Receptor 2 (*MCHR2*), Unc-5 Netrin Receptor D (*UNC5D*), or Mastermind Like Transcriptional Coactivator 2 (*MAML2*), showed high values in the 0 to 5 scale assigned by DREAMgenics (DG) value (integrated score of several predictive algorithms [34], see supplemental methods for details) used to predict deleterious mutations and, therefore, they might constitute new driver events in chondrosarcoma (Figure 3E and Figure 4B). Another group of five mutated genes was filtered from variations included in Clusters 2–4, which become enriched in CDS17 and T-CDS17 cells. The most notably alterations in this group was the mutations p.S215R and p.P72R in *TP53*, previously detected by Sanger sequencing (Figure 2A), which was present in both alleles due to a copy-neutral (CN) loss of heterozygosity (LOH) event in chromosome 17 as detailed below. Most relevant variations emerging in T-CDS17 cells (Clusters 5 and 6) included mutations in 20 genes. Among them, a SNV in F-Box and WD Repeat Domain Containing 5 (*FBXW5*) is the only variation previously reported in COSMIC in tumor types different to chondrosarcoma (Figure 3E). Also, the set of variants of the tumor sample that were lost in CDS17 and T-CDS17 cells (Clusters 10–14) included a mutation in Kinesin Family Member 21A (*KIF21A*) previously reported in COSMIC for other types of tumor and other 13 unreported mutations. Finally, three mutations were filtered from variations contained in Clusters 8 and 9, which appear in a small fraction of CDS17 cells and disappeared again in T-CDS17 cells (Figure 3E). Besides the above described changes in somatic mutations, a major consequence of in vitro and in vivo growth of tumor cells was the emergence of structural alterations and copy number variants (CNV) leading to numerous LOH events in many mutations of CDS17 and T-CDS17 cells (Figure 4A,B). Most relevant structural alterations detected in CDS17 and T-CDS17 cells included a CN-LOH affecting chromosome 17, which would explain the homozygous mutations of the *TP53* gene (p.S215R and p.P72R) commented above. Analysis of variant frequencies in this chromosome showed that the CN-LOH affected the whole chromosome and is detected in virtually all cells, as indicated by the disappearance of almost all intermediate frequencies in CDS17 and T-CDS17 cells (Figure 4B,C). Similar CN-LOH events were detected in chromosome 16, although in this case the structural variation affects to only a subset of the cells, as seen by the shift in the intermediate frequencies of variants in CDS17 and T-CDS-17 cells as compared to normal and tumor samples. Noteworthy, the intermediate frequencies shift also indicated that the subpopulation presenting the CN-LOH in chromosome 16 increased in T-CDS17 as compared to CDS-17 cells (Figure 4B,C). Besides these CN variations, other LOH events were due to CNV in several chromosomes. Thus, a copy of chromosome 18 was lost in a subpopulation of CDS-17 cells and in the whole population of non-synonymous mutations undergoing LOH in each sample. COSMIC status, DG algorithm value, and mutation type are indicated.

After filtering LOH events using the criteria described above for somatic variants and including also other LOH variants with a recurrence in the COSMIC database higher than 10, we selected 24 mutations that underwent LOH in CDS17, 17 LOH events which emerged in T-CDS-17 and 5 mutations which were detected as LOH variations in CDS17 but not in T-CDS17 (Figure 4D). Altogether, these data indicate that these cell lines kept the most relevant driver mutations present in the founder clone of the tumor sample. In addition, the adaptation of tumor cells to in vitro cell culture and in vivo growth is accompanied by the gain/loss of additional point mutations and structural variants affecting different subclones of the cell lines.

### 3.4. Analysis of CSC Subpopulations in New Chondrosarcoma Cell Lines

**Figure 5:**
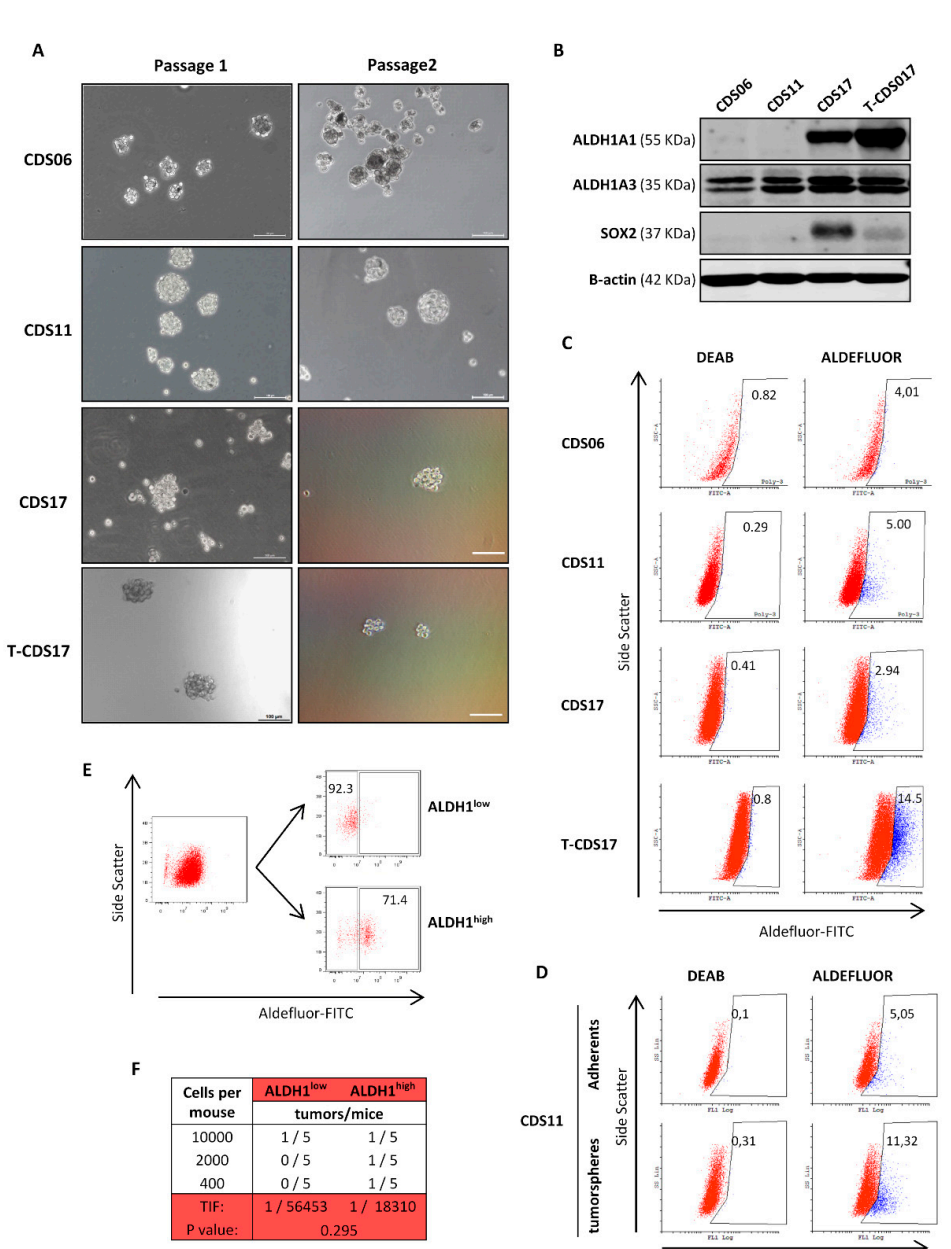
Characterization of cancer stem cell (CSC) subpopulations in chondrosarcoma cell lines. (**A**) Representative images of tumorspheres formed from the indicated cell lines in two successive passages. Scale bar = 100 µm. (**B**) Protein levels of Aldehyde Dehydrogenase 1 Family Member-A1 (ALDH1A1), -A3 (ALDH1A3), and Sex Determining Region Y-Box 2 (SOX2) in the indicated cell lines. β-actin levels were used as a loading control. (**C**) Aldefluor assay showing the activity of ALDH1 in the indicated cell lines. ALDH1 activity was blocked with the specific inhibitor N,N-diethylaminobenzaldehyde (DEAB) to establish the basal levels. (**D**) Comparison of Aldefluor activity in adherent and tumorsphere cultures of CDS11 cells. (**E**) Flow cytometry cell sorting of Aldefluor high (ALDH1^high^) and low (ALDH1^low^) populations in T-CDS17 cells. (**F**) Limiting dilution assay to evaluate tumor initiation frequency (TIF) of ALDH1^high^ and ALDH1^low^ T-CDS17 cells. The number of mice that grew tumors after 4 months and the total number of inoculated mice for each condition is indicated. TIF was calculated using ELDA software.

All the cell lines (CDS06, CDS11, CDS17, and T-CDS17) could be cultured as tumorspheres for at least two passages showing sphere forming frequencies between 0.20% and 0.28% in the first passage and between 0.1% and 0.16% in the second passage (Figure 5A and Table 1). Regarding the expression of CSC-related factors, CDS17 and T-CDS17 cells displayed high/medium levels of SOX2, while we could not detect SOX2 expression in CDS06 or CDS11 lines (Figure 5B). A similar pattern of expression was detected for ALDH1A1, while all the cell lines expressed ALDH1A3 (Figure 5B). According to the important contribution of these ALDH1A3 isoform to the Aldefluor activity [13], we detected Aldefluor positive cells in all lines in percentages ranging from 2.94% to 14.5% of the cells (Figure 5C). As previously shown in other sarcoma models [13], tumorsphere cultures of the CDS11 cell line are enriched in Aldefluor activity (Figure 5D). Furthermore, to confirm that Aldefluor could be used as a bone fide CSC marker in chondrosarcoma cell lines, we sorted T-CDS17 cells displaying high (ALDH^high^) and low (ALDH^low^) Aldefluor activity (Figure 5E) and inoculated several low cellular doses (LDA assays) subcutaneously into immunodeficient mice. We found low incidences of tumor growth after the inoculation of these cellular doses (we detected tumors in 4 out of 30 mice). In any case, ALDH^low^ cells developed only one tumor in the series where the higher number of cells was inoculated, whereas ALDH^high^ cells were able to develop three tumors, one per series. Using ELDA software to calculate the tumor initiation frequency of each population, we found that the ALDH^high^ subpopulation is three times more enriched in CSCs than the ALDH^low^ subpopulation (Figure 5F). Although, due to relatively low tumorigenicity of these cells when injected at low cellular doses, these results do not reach statistical significance, they clearly suggest that ALDH activity could be a suitable stem cell marker in these cells.

### 3.5. Invasion Ability of Chondrosarcoma Cell Lines

**Figure 6:**
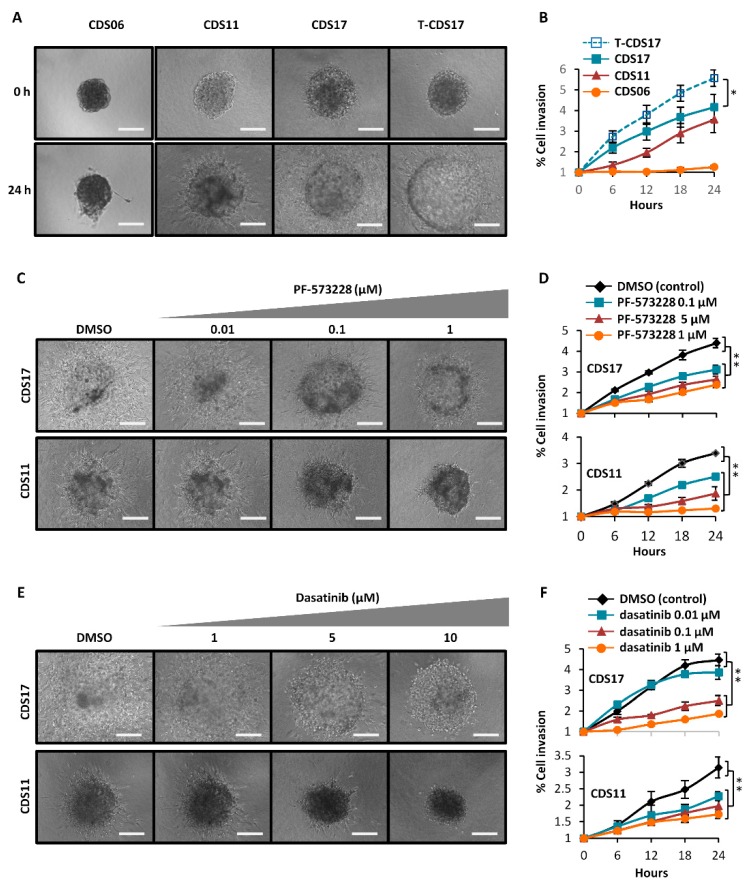
Invasive ability of chondrosarcoma cell lines. (**A**,**B**) Analysis of the invasive properties of the indicated cell lines using 3D spheroid invasion assays. Representative images of the 3D invading spheroids at the initial and final time points (**A**) and quantification of the invasive area (**B**) are presented. (**C**,**F**) Effect of increasing concentrations of PF-573228 (**C**,**D**) or dasatinib (**E**,**F**) on the invasive ability of CDS11 and CDS17 cells. Representative images of the 3D spheroids treated with the indicated concentrations of PF-573228 (**C**) or dasatinib (**E**) for 24 h and quantification of the invasive area after each treatment (**D**,**F**) are presented. Scale bars = 200 µm. Error bars represent the SD, and asterisks indicate statistically significant differences between the indicated series (* *p* < 0.05; ** *p* < 0.01; two-sided Student’s t-test). (DMSO: Dimethyl sulfoxide).

Using live cell time-lapse microscopy we found that spheroids of CDS11, CDS17, and T-CDS17—but not CDS06 cells—were able to invade 3D collagen matrices (Figure 6A,B). In accordance with its enhanced aggressiveness, T-CDS17 also displayed a significantly increased invasive potential when compared to the parental CDS17 cell line.

Deregulated SRC/FAK (Steroid receptor coactivator/Focal adhesion kinase) signaling is related to enhanced migration and invasion in many types of tumors and we previously found that several sarcoma models may invade through a mechanism depending on SRC/FAK signaling [30]. Here, we found that both the SRC inhibitor dasatinib or the FAK inhibitor PF-573228 were able to dose-dependently inhibit cell invasion, thus indicating that this mechanism is also mediating invasion in chondrosarcoma cell lines (Figure 6C–F).

## 4. Discussion

Chondrosarcomas are inherently resistant to conventional treatments and a range of new therapies aimed to target specific alterations are being currently tested [9,11,12]. Among them, the use of IDH inhibitors have not proved preclinical anti-tumor activity [49] and clinical trials including chondrosarcoma patients have not yet reported positive results [12].

Other therapeutic strategies that are being tested at clinical level include the targeting of signaling pathways controlled by hedgehog, SRC or PI3K/AKT/mTOR, as well as histone deacetylase inhibitors or anti-angiogenic agents, with only a few of them reporting partially encouraging results [12,50,51]. Altogether, there is an urgent need for more research aimed to find and test new therapies for advanced or unresectable chondrosarcomas.

Cell lines are easy to culture, relatively inexpensive and amenable to high-throughput screening models that have guided advances in cancer research for decades. Despite limitations, such as the accumulation of new mutations after endless in vitro culture [17], international studies reported that large panels of cell lines recapitulated the most relevant alterations of original tumors and, when using a relevant number of well characterized cell lines of a given cancer subtype, they were useful models to predict anti-cancer drug sensitivity and clinical outcomes [15,52,53,54]. These studies have contributed to retrieve the interest in substituting veteran endless passaged cell lines and replace them with new patient-derived cell lines which should be tagged with clinical information and include genomic characterization of both the cell line and the patient samples. These cell line models would complement more sophisticated models such as organoids or patient-derived xenografts [55].

Here we present four new chondrosarcoma cell lines with related clinical information and genetic characterization of the most common alterations (mutations in *IDH1*, *IDH2*, *TP53*, and *PI3KCA* and copy number variants in *MDM2* and *CDKN2*). All the alterations detected were also found in the original tumors with the exception of the *TP53* mutations and *MDM2* amplification found in CDS17 and T-CDS17 cell lines but not in tumor samples. This finding suggests that the loss of functional p53 could be a mechanism of adaptation to in vitro culture in chondrosarcoma cells. Our cell lines expand the panel of available chondrosarcoma cell lines (overviewed in Table S3). Of note, our study provides the first cell line (CDS06) derived from a secondary chondrosarcoma associated to a previous osteochondroma. In addition we add new dedifferentiated cchondrosarcoma cell lines with *IDH2* mutations to only one reported so far. Interestingly, none of previously published dedifferentiated lines described mutations in *IDH1* (Table S3).

Relevant for possible future applications in cancer research, three of the cell lines (CDS11, CDS17, and T-CDS17) were able to initiate tumors in vivo resembling the histology of the patient sample after inoculation in heterotopic and/or orthotopic sites. Some of these cell lines were also able to invade 3D matrices and all of them showed CSC-related features such as the ability to grow as tumorspheres or the presence of subpopulations with ALDH^high^ activity. Related to this, it has been previously shown that sarcoma cells increased their stemness and tumorigenic potential after being grown in mice [13,56]. Therefore cell line/xenograft line tandems, like the one formed by CDS17 and T-CDS17, constitute valuable models for studying/tracking cancer stem cells subpopulations during tumor progression [13]. These studies point ALDH1 as a relevant CSC-associated factor in different types of sarcoma [13,56]. Similarly, we found that the T-CDS17 cell line showed increased ALDH1 expression and activity, enhanced invasive ability and increased in vivo tumorigenic potential than its parental CDS17 cell line. Moreover, our results also suggest a role for ALDH1 as CSC marker in chondrosarcoma.

Our work also includes a WES analysis of CDS17 and T-CDS17 cells lines together with normal and tumor samples from the patient. This is the first time that a chondrosarcoma cell line and matched patient samples include such a level of genomic characterization. This analysis allowed us to know how the cell lines resemble the genomic diversity of the original tumor and also to track the genomic evolution of tumor cells during in vitro and in vivo growth. We found that the putative founder clone, that including a higher number of mutations and is present in all tumor cells, remains unaltered after in vitro cell culture (CDS17 cells) and in vivo growth (T-CDS17 cells). This clone includes previously known mutations, such as that previously found by Sanger sequencing in *IDH2* (R172G) [2,4] or mutations in *COL2A1* (G939Wfs*5 and P668Lfs*120) [5,6], as well as other unreported non-synonymous mutations presenting high scores in impact prediction algorithms, such as *VNN2*, *CACNA1D*, *MCHR2*, *UNC5D*, or *MAML2*, which possibly contribute to chondrosarcoma progression must be studied in detail.

Other mutations affecting different subclones appear or disappear during the adaptation of cells to in vitro/in vivo growth. Although not widely described for sarcomas, this genomic drift is in line with previous studies in other cancer types [57]. An important phenomenon related to the adaptation to growth conditions is the emergence of structural alterations in several chromosomes in CDS17 and T-CDS17 cells. The most relevant change was the CN-LOH affecting the chromosome 17 and responsible for appearance of homozygous mutations in *TP53*. Importantly, alterations in *TP53* were associated with aggressive behavior of chondrosarcomas [58] and similar LOH affecting chromosome 17 was detected in high grade chondrosarcomas [59]. Therefore, despite not being present in the original tumor, the CN-LOH in chromosome 17 detected in CDS17 and T-CDS17 resembles a naturally occurring mechanism for increasing aggressiveness in chondrosarcomas. Other structural variants, such as those detected chromosomes like 16, 18, or 20, affected a sequentially increased cell population in CDS17 and T-CDS17 cells. Given that T-CDS17 cells are more aggressive tan CDS17 cells, some of these structural alterations could be involved in the gain of malignancy in T-CDS17 cells. Whether these alterations occur also in patients during tumor progression or are only due to the adaptation of tumor cells to grow ex-vivo remains to be studied.

Although we could not be completely sure that all the genetic differences observed between CDS17 and T-CDS17 cell lines were due to the in vivo growth in mice and not to the ex-vivo expansion of T-CDS17, the fact that the genomic drift previously observed in patient-derived cell lines were mainly restricted to the first few passages [57] when the adaptation of cells to the in vitro growth conditions occurs, suggests that the new set of genetic alterations detected in T-CDS17 most likely emerged during the in vivo growth phase.

## 5. Conclusions

In summary, this study provides new patient-derived chondrosarcoma cell lines with clinical and genetic information from patients. These cell lines are suitable for studying CSC subpopulations and to generate in vivo models for this disease. Furthermore, a pioneering genomic analysis using a cell line (CDS17)/xenograft line (T-CDS-17) tandem model confirmed that these cell lines kept the most relevant mutations of the original tumor and described the genetic drift process that tumor cells underwent during the adaptation to in vitro and in vivo growth.

## Figures and Tables

**Table 1:** Functional characterization of chondrosarcoma cell lines.

Cell Line	Chondrosar. Subtype	Anchorage Independ. Growth ^§^	Passage *	Tumorsphere Growth (% Frequency ± SD)		In Vivo Tumor Growth **			Aldefluor Assay (%)	Invasion ^‡^
						Subcutaneous		Intra-Bone		
				1st Tumorsph. Passage	2nd Tumorsph. Passage	Tumor Growth (Tumors/Mice)	Mean Volume (mm^3^ ± SD)	Tumor Growth (Tumors/Mice)		
**CDS06**	Secondary	Yes	20	Yes (0.24 ± 0.10)	Yes (0.10 ± 0.05)	n/a	n/a	n/a	Yes (4.01)	No
**CDS11**	Secondary	Yes	25	Yes (0.28 ± 0.11)	Yes (0.16 ± 0.06)	Yes (3/3)	77.00 ± 10.91	n/a	Yes (5.05)	Yes
**CDS17**	Dedifferent.	Yes	<35	Yes (0.20 ± 0.08)	Yes (0.10 ± 0.01)	Yes (3/3)	198.27 ± 5.11	Yes (1/2)	Yes (2.94)	Yes
**T-CDS17**	Dedifferent.	Yes	<35	Yes (0.26 ± 0.08)	Yes (0.11 ± 0.01)	Yes (3/3)	350.32 ± 39.45 ¶	Yes (2/2)	Yes (14.50)	Yes

(§) Ability to growth forming colonies in embedded in soft agar. (*) number of passages reached so far in adherent cultures. (**) Tumor growth was follow for 1 and 2.5 months in subcutaneous and intra-bone experiments respectively. (‡) Ability of 3D spheroids to invade collagen matrices. (¶) There is a significant difference between the volumes of tumors generated by CDS17 and T-CDS17 cells (*p* = 0.043; two side Student’s *t*-test. SD: Standard Deviation).

## Supplementary Materials

The following are available online at https://www.mdpi.com/2077-0383/8/4/455/s1, Supplemental Materials and Methods, Figure S1: Computerized tomography scan (CT) at day 50 after intra-bone inoculation, Figure S2: WES analysis of chondrosarcoma cell lines, Table S1: Patient and tumor characteristics, Table S2: STR analysis for the indicated patient-derived primary cell lines and the corresponding tumor tissue of origin.

## References

[1] Fletcher C., Bridge J., Hogendoorn P., Mertens F. (2013). WHO Classification of Tumours of Soft Tissue and Bone. Pathology and Genetics of Tumours of Soft Tissue and Bone.

[2] Amary M.F., Bacsi K., Maggiani F., Damato S., Halai D., Berisha F., Pollock R., O’Donnell P., Grigoriadis A., Diss T. (2011). IDH1 and IDH2 mutations are frequent events in central chondrosarcoma and central and periosteal chondromas but not in other mesenchymal tumours. J. Pathol..

[3] Lu C., Venneti S., Akalin A., Fang F., Ward P.S., Dematteo R.G., Intlekofer A.M., Chen C., Ye J., Hameed M. (2013). Induction of sarcomas by mutant IDH2. Genes Dev..

[4] Tinoco G., Wilky B.A., Paz-Mejia A., Rosenberg A., Trent J.C. (2015). The biology and management of cartilaginous tumors: A role for targeting isocitrate dehydrogenase. Am. Soc. Clin. Oncol. Educ. Book.

[5] Tarpey P.S., Behjati S., Cooke S.L., Van Loo P., Wedge D.C., Pillay N., Marshall J., O’Meara S., Davies H., Nik-Zainal S. (2013). Frequent mutation of the major cartilage collagen gene COL2A1 in chondrosarcoma. Nat. Genet..

[6] Totoki Y., Yoshida A., Hosoda F., Nakamura H., Hama N., Ogura K., Fujiwara T., Arai Y., Toguchida J., Tsuda H. (2014). Unique mutation portraits and frequent COL2A1 gene alteration in chondrosarcoma. Genome Res..

[7] Schrage Y.M., Lam S., Jochemsen A.G., Cleton-Jansen A.M., Taminiau A.H., Hogendoorn P.C., Bovee J.V. (2009). Central chondrosarcoma progression is associated with pRb pathway alterations: CDK4 down-regulation and p16 overexpression inhibit cell growth in vitro. J. Cell. Mol. Med..

[8] Boehme K.A., Schleicher S.B., Traub F., Rolauffs B. (2018). Chondrosarcoma: A Rare Misfortune in Aging Human Cartilage? The Role of Stem and Progenitor Cells in Proliferation, Malignant Degeneration and Therapeutic Resistance. Int. J. Mol. Sci..

[9] Bovee J.V., Cleton-Jansen A.M., Taminiau A.H., Hogendoorn P.C. (2005). Emerging pathways in the development of chondrosarcoma of bone and implications for targeted treatment. Lancet Oncol..

[10] Brown H.K., Schiavone K., Gouin F., Heymann M.F., Heymann D. (2018). Biology of Bone Sarcomas and New Therapeutic Developments. Calcif. Tissue Int..

[11] David E., Blanchard F., Heymann M.F., De Pinieux G., Gouin F., Redini F., Heymann D. (2011). The Bone Niche of Chondrosarcoma: A Sanctuary for Drug Resistance, Tumour Growth and also a Source of New Therapeutic Targets. Sarcoma.

[12] Polychronidou G., Karavasilis V., Pollack S.M., Huang P.H., Lee A., Jones R.L. (2017). Novel therapeutic approaches in chondrosarcoma. Future Oncol..

[13] Martinez-Cruzado L., Tornin J., Santos L., Rodriguez A., Garcia-Castro J., Moris F., Rodriguez R. (2016). Aldh1 Expression and Activity Increase During Tumor Evolution in Sarcoma Cancer Stem Cell Populations. Sci. Rep..

[14] Tirino V., Desiderio V., Paino F., De Rosa A., Papaccio F., Fazioli F., Pirozzi G., Papaccio G. (2011). Human primary bone sarcomas contain CD133+ cancer stem cells displaying high tumorigenicity in vivo. FASEB J..

[15] Goodspeed A., Heiser L.M., Gray J.W., Costello J.C. (2016). Tumor-Derived Cell Lines as Molecular Models of Cancer Pharmacogenomics. Mol. Cancer Res..

[16] McDermott U. (2018). Cancer cell lines as patient avatars for drug response prediction. Nat. Genet..

[17] Wilding J.L., Bodmer W.F. (2014). Cancer cell lines for drug discovery and development. Cancer Res..

[18] Calabuig-Farinas S., Benso R.G., Szuhai K., Machado I., Lopez-Guerrero J.A., de Jong D., Peydro A., San Miguel T., Navarro L., Pellin A. (2012). Characterization of a new human cell line (CH-3573) derived from a grade II chondrosarcoma with matrix production. Pathol. Oncol. Res..

[19] Farges M., Mazeau C., Gioanni J., Ettore F., Denovion H., Schneider M. (1997). Establishment and characterization of a new cell line derived from a human chondrosarcoma. Oncol. Rep..

[20] Gil-Benso R., Lopez-Gines C., Lopez-Guerrero J.A., Carda C., Callaghan R.C., Navarro S., Ferrer J., Pellin A., Llombart-Bosch A. (2003). Establishment and characterization of a continuous human chondrosarcoma cell line, ch-2879: Comparative histologic and genetic studies with its tumor of origin. Lab. Investig..

[21] Jagasia A.A., Block J.A., Qureshi A., Diaz M.O., Nobori T., Gitelis S., Iyer A.P. (1996). Chromosome 9 related aberrations and deletions of the CDKN2 and MTS2 putative tumor suppressor genes in human chondrosarcomas. Cancer Lett..

[22] Kalinski T., Krueger S., Pelz A.F., Wieacker P., Hartig R., Ropke M., Schneider-Stock R., Dombrowski F., Roessner A. (2005). Establishment and characterization of the permanent human cell line C3842 derived from a secondary chondrosarcoma in Ollier’s disease. Virchows Arch..

[23] Kudawara I., Araki N., Myoui A., Kato Y., Uchida A., Yoshikawa H. (2004). New cell lines with chondrocytic phenotypes from human chondrosarcoma. Virchows Arch..

[24] Kudo N., Ogose A., Hotta T., Kawashima H., Gu W., Umezu H., Toyama T., Endo N. (2007). Establishment of novel human dedifferentiated chondrosarcoma cell line with osteoblastic differentiation. Virchows Arch..

[25] Kunisada T., Miyazaki M., Mihara K., Gao C., Kawai A., Inoue H., Namba M. (1998). A new human chondrosarcoma cell line (OUMS-27) that maintains chondrocytic differentiation. Int. J. Cancer.

[26] Monderer D., Luseau A., Bellec A., David E., Ponsolle S., Saiagh S., Bercegeay S., Piloquet P., Denis M.G., Lode L. (2013). New chondrosarcoma cell lines and mouse models to study the link between chondrogenesis and chemoresistance. Lab. Investig..

[27] van Oosterwijk J.G., de Jong D., van Ruler M.A., Hogendoorn P.C., Dijkstra P.D., van Rijswijk C.S., Machado I., Llombart-Bosch A., Szuhai K., Bovee J.V. (2012). Three new chondrosarcoma cell lines: One grade III conventional central chondrosarcoma and two dedifferentiated chondrosarcomas of bone. BMC Cancer.

[28] Tornin J., Martinez-Cruzado L., Santos L., Rodriguez A., Nunez L.E., Oro P., Hermosilla M.A., Allonca E., Fernandez-Garcia M.T., Astudillo A. (2016). Inhibition of SP1 by the mithramycin analog EC-8042 efficiently targets tumor initiating cells in sarcoma. Oncotarget.

[29] Martinez-Cruzado L., Tornin J., Rodriguez A., Santos L., Allonca E., Fernandez-Garcia M.T., Astudillo A., Garcia-Pedrero J.M., Rodriguez R. (2017). Trabectedin and Campthotecin Synergistically Eliminate Cancer Stem Cells in Cell-of-Origin Sarcoma Models. Neoplasia.

[30] Tornin J., Hermida-Prado F., Padda R.S., Gonzalez M.V., Alvarez-Fernandez C., Rey V., Martinez-Cruzado L., Estupinan O., Menendez S.T., Fernandez-Nevado L. (2018). FUS-CHOP Promotes Invasion in Myxoid Liposarcoma through a SRC/FAK/RHO/ROCK-Dependent Pathway. Neoplasia.

[31] Estupinan O., Santos L., Rodriguez A., Fernandez-Nevado L., Costales P., Perez-Escuredo J., Hermosilla M.A., Oro P., Rey V., Tornin J. (2018). The multikinase inhibitor EC-70124 synergistically increased the antitumor activity of doxorubicin in sarcomas. Int. J. Cancer.

[32] Rubio R., Abarrategi A., Garcia-Castro J., Martinez-Cruzado L., Suarez C., Tornin J., Santos L., Astudillo A., Colmenero I., Mulero F. (2014). Bone environment is essential for osteosarcoma development from transformed mesenchymal stem cells. Stem Cells.

[33] Bolger A.M., Lohse M., Usadel B. (2014). Trimmomatic: A flexible trimmer for Illumina sequence data. Bioinformatics.

[34] Cabanillas R., Dineiro M., Castillo D., Pruneda P.C., Penas C., Cifuentes G.A., de Vicente A., Duran N.S., Alvarez R., Ordonez G.R. (2017). A novel molecular diagnostics platform for somatic and germline precision oncology. Mol. Genet. Genomic Med..

[35] Choi Y., Sims G.E., Murphy S., Miller J.R., Chan A.P. (2012). Predicting the functional effect of amino acid substitutions and indels. PLoS ONE.

[36] Chun S., Fay J.C. (2009). Identification of deleterious mutations within three human genomes. Genome Res..

[37] Davydov E.V., Goode D.L., Sirota M., Cooper G.M., Sidow A., Batzoglou S. (2010). Identifying a high fraction of the human genome to be under selective constraint using GERP++. PLoS Comput. Biol..

[38] Deshwar A.G., Vembu S., Yung C.K., Jang G.H., Stein L., Morris Q. (2015). PhyloWGS: Reconstructing subclonal composition and evolution from whole-genome sequencing of tumors. Genome Biol..

[39] Dong C., Wei P., Jian X., Gibbs R., Boerwinkle E., Wang K., Liu X. (2015). Comparison and integration of deleteriousness prediction methods for nonsynonymous SNVs in whole exome sequencing studies. Hum. Mol. Genet..

[40] Jagadeesh K.A., Wenger A.M., Berger M.J., Guturu H., Stenson P.D., Cooper D.N., Bernstein J.A., Bejerano G. (2016). M-CAP eliminates a majority of variants of uncertain significance in clinical exomes at high sensitivity. Nat. Genet..

[41] Kumar P., Henikoff S., Ng P.C. (2009). Predicting the effects of coding non-synonymous variants on protein function using the SIFT algorithm. Nat. Protoc..

[42] Li H., Handsaker B., Wysoker A., Fennell T., Ruan J., Homer N., Marth G., Abecasis G., Durbin R., Genome Project Data Processing S. (2009). The Sequence Alignment/Map format and SAMtools. Bioinformatics.

[43] Miller C.A., McMichael J., Dang H.X., Maher C.A., Ding L., Ley T.J., Mardis E.R., Wilson R.K. (2016). Visualizing tumor evolution with the fishplot package for R. BMC Genom..

[44] Puente X.S., Pinyol M., Quesada V., Conde L., Ordonez G.R., Villamor N., Escaramis G., Jares P., Bea S., Gonzalez-Diaz M. (2011). Whole-genome sequencing identifies recurrent mutations in chronic lymphocytic leukaemia. Nature.

[45] Reva B., Antipin Y., Sander C. (2011). Predicting the functional impact of protein mutations: Application to cancer genomics. Nucleic Acids Res..

[46] Schwarz J.M., Cooper D.N., Schuelke M., Seelow D. (2014). MutationTaster2: Mutation prediction for the deep-sequencing age. Nat. Methods.

[47] Shihab H.A., Gough J., Mort M., Cooper D.N., Day I.N., Gaunt T.R. (2014). Ranking non-synonymous single nucleotide polymorphisms based on disease concepts. Hum. Genom..

[48] European Nucleotide Archive repository. http://www.ebi.ac.uk/ena/data/view/PRJEB31233.

[49] Suijker J., Oosting J., Koornneef A., Struys E.A., Salomons G.S., Schaap F.G., Waaijer C.J., Wijers-Koster P.M., Briaire-de Bruijn I.H., Haazen L. (2015). Inhibition of mutant IDH1 decreases D-2-HG levels without affecting tumorigenic properties of chondrosarcoma cell lines. Oncotarget.

[50] Campbell V.T., Nadesan P., Ali S.A., Wang C.Y., Whetstone H., Poon R., Wei Q., Keilty J., Proctor J., Wang L.W. (2014). Hedgehog pathway inhibition in chondrosarcoma using the smoothened inhibitor IPI-926 directly inhibits sarcoma cell growth. Mol. Cancer Ther..

[51] Heymann D., Redini F. (2013). Targeted therapies for bone sarcomas. Bonekey Rep..

[52] Barretina J., Caponigro G., Stransky N., Venkatesan K., Margolin A.A., Kim S., Wilson C.J., Lehar J., Kryukov G.V., Sonkin D. (2012). The Cancer Cell Line Encyclopedia enables predictive modelling of anticancer drug sensitivity. Nature.

[53] Iorio F., Knijnenburg T.A., Vis D.J., Bignell G.R., Menden M.P., Schubert M., Aben N., Goncalves E., Barthorpe S., Lightfoot H. (2016). A Landscape of Pharmacogenomic Interactions in Cancer. Cell.

[54] Lee J.K., Liu Z., Sa J.K., Shin S., Wang J., Bordyuh M., Cho H.J., Elliott O., Chu T., Choi S.W. (2018). Pharmacogenomic landscape of patient-derived tumor cells informs precision oncology therapy. Nat. Genet..

[55] Ledford H. (2016). US cancer institute to overhaul tumour cell lines. Nature.

[56] Golan H., Shukrun R., Caspi R., Vax E., Pode-Shakked N., Goldberg S., Pleniceanu O., Bar-Lev D.D., Mark-Danieli M., Pri-Chen S. (2018). In Vivo Expansion of Cancer Stemness Affords Novel Cancer Stem Cell Targets: Malignant Rhabdoid Tumor as an Example. Stem Cell Rep..

[57] Ben-David U., Ha G., Tseng Y.Y., Greenwald N.F., Oh C., Shih J., McFarland J.M., Wong B., Boehm J.S., Beroukhim R. (2017). Patient-derived xenografts undergo mouse-specific tumor evolution. Nat. Genet..

[58] Oshiro Y., Chaturvedi V., Hayden D., Nazeer T., Johnson M., Johnston D.A., Ordonez N.G., Ayala A.G., Czerniak B. (1998). Altered p53 is associated with aggressive behavior of chondrosarcoma: A long term follow-up study. Cancer.

[59] Yamaguchi T., Toguchida J., Wadayama B., Kanoe H., Nakayama T., Ishizaki K., Ikenaga M., Kotoura Y., Sasaki M.S. (1996). Loss of heterozygosity and tumor suppressor gene mutations in chondrosarcomas. Anticancer Res..

