# Supplementary material for: New Chondrosarcoma Cell Lines with Preserved Stem Cell Properties to Study the Genomic Drift During In Vitro/In Vivo Growth
Source: J Clin Med. 2019 Apr 4;8(4):455. doi: 10.3390/jcm8040455 (PMC6518242; doi:10.3390/jcm8040455)
Supplement: Supplementary file 1 [file jcm-08-00455-s001.zip › Supplemental Information - Rey et al.pdf]

# NEW CHONDROSARCOMA CELL LINES WITH PRESERVED STEM CELL PROPERTIES TO STUDY THE GENOMIC DRIFT DURING IN VITRO / IN VIVO GROWTH

**Running Title: New preclinical models of chondrosarcoma**

Veronica Rey[1,2 *], Sofia T. Menendez[1,2,3 *], Oscar Estupiñan[1,2,3], Aida Rodriguez[1], Laura Santos[1], Juan Tornín[1], Lucía Martínez-Cruzado[1], David Castillo[4], Gonzalo Ordoñez[4], Serafín Costilla[5], Carlos Alvarez-Fernández[6], Aurora Astudillo[7], Alejandro Braña[8] and Rene Rodriguez[1, 2,3 §]

[1]Hospital Universitario Central de Asturias - Instituto de Investigación Sanitaria del Principado de Asturias, Oviedo, Spain; [2]Instituto Universitario de Oncología del Principado de Asturias, Oviedo, Spain. [3]CIBER en oncología (CIBERONC), Madrid, Spain. [4]Disease Research And Medicine (DREAMgenics) S. L., Oviedo, Spain. [5]Servicio de Radiología, [6]Servicio de Oncología Médica, [7]Servicio de Anatomía Patológica and [8]Servicio de Traumatología of the Hospital Universitario Central de Asturias, Oviedo, Spain.

## SUPPLEMENTAL INFORMATION

## SUPPLEMETAL MATERIALS AND METHODS

### Short Tandem Repeat (STR) analysis.

Comparison of the identity of the cell lines with the original patient sample was done at Sequencing Core Facilities of the University of Oviedo (Oviedo, Spain) by STR analysis of the corresponding genomic DNA using the AmpFISTR® Identifiler® PCR Amplification Kit (Thermofisher, Waltham, MA) according to the manufacturer's instructions.

### Western blot antibodies

The antibodies used in Western blot analysis were as follows: anti-ALDH1A1 [(ab105920), 1:1,000 dilution] from Abcam (Cambridge, UK); anti-ALDH1A3 [(AP7847a), 1:50 dilution] from Abgent (San Diego, CA); anti-SOX2 [(PA1-094), 1:1,000 dilution] from Thermo Fisher (Waltham, MA); and anti-β-Actin [(A-1978), 1:20,000 dilution] from Sigma.

**Three-dimensional spheroid invasion assay.**

Cell spheroids were prepared using a hanging drop protocol as previously described [30]. Cell invasion in the presence or not of dasatinib or PF-573228 (Selleckchem, Houston, TX) was monitored using a Zeiss Cell Observer Live Imaging microscope (Zeiss, Thornwood, NY) coupled with a $CO_2$ and temperature-maintenance system. Time-lapse images were acquired every 15 minutes during 24 hours using a Zeiss AxioCam MRc camera. The invasive area was determined by calculating the difference between the final area (t = 24 h) and the initial area (t = 0 h) using image J analysis program, and data were normalized to the control cells. 3 independent experiments including 4 replicates for each condition were performed.

**Primers for Sanger sequencing**

PCR reactions for Sanger sequencing were carried out using the forward and reverse primers detailed below:

| Gene | Exon | Codon | Forward (Fw) 5' – 3' | Reverse (Rv) 5' – 3' | Reference Sequence |
|---|---|---|---|---|---|
| IDH1 | 4 | 101 to 133 | CACCAAATGGCACCATACGA | CAATTTCATACCTTGCTTAATGGG | NM_001282387 |
| IDH2 | 4 | 126 to 178 | ATTCTGGTTGAAAGATGGCG | AAGTCTGTGGCCTTGTACTG | NM_002168 |
| PIK3CA | 20 | 997 to 1068 | TATTCGACAGCATGCCAATC | CCTATGCAATCGGTCTTTGC | NM_006218 |
| TP53 | 4 | 33 to 125 | TGAGGACCTGGTCCTCTGAC | GATACGGCCAGGCATTGAAG | NM_001126114 |
| | 6 | 187 to 224 | GCCTCTGATTCCTCACTGAT | GCCACTGACAACCACCCTTA | |

**Bioinformatic analysis of WES data**

WES results were processed using the bioinformatics software HD Genome One (DREAMgenics, Oviedo, Spain), certified with IVD/CE-marking. The exome analysis pipeline included the following steps:

*Quality control and Alignment*

Quality controls were performed using FastQC (http://www.bioinformatics.babraham.ac.uk/projects/fastqc/). Removal of low quality bases, adapters and other technical sequences was conducted with Trimmomatic [33]. BWA-mem was then employed for alignment to the human reference genome (GRCh37/hg19), generating sorted BAM Files with SAMtools [42]. Finally, optical and PCR duplicates were eliminated using Picard (http://broadinstitute.github.io/picard/).

*Somatic Variant Calling*

A variation of Sidrón algorithm was employed for the identification [44] of SNVs and indels, which performance has been previously described [34]. Somatic variants were identified using the following parameters: total read depth ≥ 15, variant frequency ≥ 0.05, base quality ≥20, mapping quality ≥30. Mutations detected in the tumor samples were interrogated in the normal sample in order to define the somatic status of each variant.

*Variant annotation*

The following databases were consulted for variant annotation: population databases (1000 Genomes, ESP, dbSNP, ExAC), functional information (Ensembl, CCDS, RefSeq, Pfam), disease-related (Clinvar) as well as 1 score (GERP++) for evolutionary conservation of the affected nucleotide [37]. To estimate the protein impact of point mutations the following predictive algorithms were taken into account: SIFT [41], PolyPhen2 [35], PROVEAN [35], Mutation Assessor [45], Mutation Taster [46], LRT [36], MetaLR, MetaSVM [39], FATHMM, FATHMM-MKL [47] and M-CAP [40]. Their predictive score was integrated and normalized into a single scaled value (DG value), ranging from 0 (tolerated) to 5 (deleterious).

*CNV and CN-LOH identification*

The detection of CNVs and CN-LOH was performed employing exome2cnv, evaluating read depth and allelic imbalance computations. The algorithm employs a background of pooled samples processed using the same capturing protocol and sequencing technology [34,44]

*Subclonal composition and Evolution*

Subclonal reconstruction was performed with PhyloWGS [38]. For this analysis somatic variants from all tumor samples were extracted to generate a pool of somatic mutations. To correctly assess their representation, only those positions with coverage >= 15 in all samples were considered. Population evolution was then represented using the Fishplot package for R [43].

**CT and μCT analysis**

In intra-bone inoculation experiments, tumor growth was evaluated in the Preclinical Image laboratory of the University of Oviedo. At week 8 after cell inoculation mice were analyzed in a Computered Tomography (CT) system (Argus CT, Sedecal, Madrid, Spain) with an X-ray tube voltage of 45 kV and a current of 300 μA. Acquired images were reconstructed using Horos, which is a free and open source code software (FOSS) program that is distributed free of charge under the LGPL license at Horosproject.org and sponsored by Nimble Co LLC d/b/a Purview in Annapolis, MD. In addition, formol fixed samples of mice sacrificed at week 12 were analyzed in a μCT system (SkyScan 1174, Bruker, Antwerp, Belgium), with an X-ray tube voltage of 50 kV and a current of 850 μA. 0.5mm Aluminium filter was used. The scanning angular rotation was 180°, the angular increment 0.3° and the voxel resolution 11,8 μm. Data sets were reconstructed using NRecon software (provided by the manufacture) and segmented into binary images and 3D surface reconstructions were made using CTAn software (SkyScan,Bruker).

## SUPPLEMENTAL REFERENCES

30. Tornin, J.; Hermida-Prado, F.; Padda, R.S.; Gonzalez, M.V.; Alvarez-Fernandez, C.; Rey, V.; Martinez-Cruzado, L.; Estupinan, O.; Menendez, S.T.; Fernandez-Nevado, L. et al. FUS-

CHOP Promotes Invasion in Myxoid Liposarcoma through a SRC/FAK/RHO/ROCK-Dependent Pathway. Neoplasia 2018, 20, 44-56. DOI: 10.1016/j.neo.2017.11.004.

33. Bolger, A.M.; Lohse, M.; Usadel, B. Trimmomatic: a flexible trimmer for Illumina sequence data. Bioinformatics 2014, 30, 2114-2120. DOI: 10.1093/bioinformatics/btu170.

34. Cabanillas, R.; Dineiro, M.; Castillo, D.; Pruneda, P.C.; Penas, C.; Cifuentes, G.A.; de Vicente, A.; Duran, N.S.; Alvarez, R.; Ordonez, G.R. et al. A novel molecular diagnostics platform for somatic and germline precision oncology. Mol Genet Genomic Med 2017, 5, 336-359. DOI: 10.1002/mgg3.291.

35. Choi, Y.; Sims, G.E.; Murphy, S.; Miller, J.R.; Chan, A.P. Predicting the functional effect of amino acid substitutions and indels. PLoS One 2012, 7, e46688. DOI: 10.1371/journal.pone.0046688.

36. Chun, S.; Fay, J.C. Identification of deleterious mutations within three human genomes. Genome Res 2009, 19, 1553-1561. DOI: 10.1101/gr.092619.109.

37. Davydov, E.V.; Goode, D.L.; Sirota, M.; Cooper, G.M.; Sidow, A.; Batzoglou, S. Identifying a high fraction of the human genome to be under selective constraint using GERP++. PLoS Comput Biol 2010, 6, e1001025. DOI: 10.1371/journal.pcbi.1001025.

38. Deshwar, A.G.; Vembu, S.; Yung, C.K.; Jang, G.H.; Stein, L.; Morris, Q. PhyloWGS: reconstructing subclonal composition and evolution from whole-genome sequencing of tumors. Genome Biol 2015, 16, 35. DOI: 10.1186/s13059-015-0602-8.

39. Dong, C.; Wei, P.; Jian, X.; Gibbs, R.; Boerwinkle, E.; Wang, K.; Liu, X. Comparison and integration of deleteriousness prediction methods for nonsynonymous SNVs in whole exome sequencing studies. Hum Mol Genet 2015, 24, 2125-2137. DOI: 10.1093/hmg/ddu733.

40. Jagadeesh, K.A.; Wenger, A.M.; Berger, M.J.; Guturu, H.; Stenson, P.D.; Cooper, D.N.; Bernstein, J.A.; Bejerano, G. M-CAP eliminates a majority of variants of uncertain significance in clinical exomes at high sensitivity. Nat Genet 2016, 48, 1581-1586. DOI: 10.1038/ng.3703.

41. Kumar, P.; Henikoff, S.; Ng, P.C. Predicting the effects of coding non-synonymous variants on protein function using the SIFT algorithm. Nat Protoc 2009, 4, 1073-1081. DOI: 10.1038/nprot.2009.86.

42. Li, H.; Handsaker, B.; Wysoker, A.; Fennell, T.; Ruan, J.; Homer, N.; Marth, G.; Abecasis, G.; Durbin, R.; Genome Project Data Processing, S. The Sequence Alignment/Map format and SAMtools. Bioinformatics 2009, 25, 2078-2079. DOI: 10.1093/bioinformatics/btp352.

43. Miller, C.A.; McMichael, J.; Dang, H.X.; Maher, C.A.; Ding, L.; Ley, T.J.; Mardis, E.R.; Wilson, R.K. Visualizing tumor evolution with the fishplot package for R. BMC Genomics 2016, 17, 880. DOI: 10.1186/s12864-016-3195-z.

44. Puente, X.S.; Pinyol, M.; Quesada, V.; Conde, L.; Ordonez, G.R.; Villamor, N.; Escaramis, G.; Jares, P.; Bea, S.; Gonzalez-Diaz, M. et al. Whole-genome sequencing identifies

recurrent mutations in chronic lymphocytic leukaemia. Nature 2011, 475, 101-105. DOI: 10.1038/nature10113.

45. Reva, B.; Antipin, Y.; Sander, C. Predicting the functional impact of protein mutations: application to cancer genomics. Nucleic Acids Res 2011, 39, e118. DOI: 10.1093/nar/gkr407.

46. Schwarz, J.M.; Cooper, D.N.; Schuelke, M.; Seelow, D. MutationTaster2: mutation prediction for the deep-sequencing age. Nat Methods 2014, 11, 361-362. DOI: 10.1038/nmeth.2890.

47. Shihab, H.A.; Gough, J.; Mort, M.; Cooper, D.N.; Day, I.N.; Gaunt, T.R. Ranking non-synonymous single nucleotide polymorphisms based on disease concepts. Hum Genomics 2014, 8, 11. DOI: 10.1186/1479-7364-8-11.

## SUPPLEMENTAL FIGURES

**Figure S1. Computerized tomography scan (CT)** at day 50 after intra-bone inoculation of CDS17 and T-CDS17 cells in immunodeficient mice. Coronal, sagittal and axial images are shown. Compared to a control leg, intra-medullar formation of tumor bone/osteoid formation (white arrows) is shown in mice inoculated with both cell lines. In addition, a mouse inoculated with T-CDS17 cells presented an extra-medullar lesion compatible with the radiographic features of osteochondrosarcoma (orange arrows).

**Figure S2. WES analysis of chondrosarcoma cell lines.** (A) profile of SNV transitions and transvertions. (B) Analysis of variant frequencies (left panels) and CN (right panels) in the indicated chromosomes.
