# Supplementary material for: New Chondrosarcoma Cell Lines with Preserved Stem Cell Properties to Study the Genomic Drift During In Vitro/In Vivo Growth
Source: J Clin Med. 2019 Apr 4;8(4):455. doi: 10.3390/jcm8040455 (PMC6518242; doi:10.3390/jcm8040455)
Supplement: Supplementary file 1 [file jcm-08-00455-s001.zip › Rey et al - Table S1.docx]

| **Table S1**. Patient and tumor characteristics | | | | | | | | | | | | | | | |  |
| --- | --- | --- | --- | --- | --- | --- | --- | --- | --- | --- | --- | --- | --- | --- | --- | --- |
| **Cell line** | **Age*** | **Gender** | **Tobacco** | **Tumor localization** | **Chondrosarcoma subtype (previous condition)** | **Histological grade** | **TMN** | **Invasion** | | | **Pre-treatment** | | **Post-treatment** | |  |  |
|  |  |  |  |  |  |  |  | **vascular** | **lymphatic** | **neural** | |  | |  | | |
| CDS06 | 61 | female | no | pelvis | secondary (OC) | 2 | pT3N0M0 | no | no | no | | no | | N.A. | | |
| CDS11 | 61 | male | yes | scapula | secondary (OD) | 1 | pT2N0M0 | no | no | no | | no | | none | | |
| CDS17 | 49 | male | no | hemipelvis | dedifferentiated | 3 | pT1N0M0 | no | no | no | | no | | CT | | |
| T-CDS17 | ­- | ­- | ­- | CDS17- xenograft line | dedifferentiated | ­3 | ­- | ­- | ­- | ­- | | ­- | | ­- | | |
| (* )Age at diagnostic. OC: osteochondroma. OD: Ollier disease. N.A.: not available data. CT: chemotherapy. | | | | | | | | | | | | | | | |  |
