# Supplementary material for: New Chondrosarcoma Cell Lines with Preserved Stem Cell Properties to Study the Genomic Drift During In Vitro/In Vivo Growth
Source: J Clin Med. 2019 Apr 4;8(4):455. doi: 10.3390/jcm8040455 (PMC6518242; doi:10.3390/jcm8040455)
Supplement: Supplementary file 1 [file jcm-08-00455-s001.zip › Rey et al - Table S2.docx]

| **Table S2. STR analysis for the indicated patient-derived primary cell lines and the corresponding tumor tissue of origin** | | | | | | | |
| --- | --- | --- | --- | --- | --- | --- | --- |
| **locus name** | **CDS06-cell line** | **CDS06-tumor tissue** | **CDS11-cell line** | **CDS11-tumor tissue** | **CDS17-cell line** | **T-CDS17-cell line** | **CDS17-tumor tissue** |
| **D7S820** | 8,10 | 8,10 | 11,12 | 11,12 | 9,10 | 9,10 | 9,10 |
| **CSF1PO** | 10,12 | 10,12 | 11,11 | 11,11 | 11,11 | 11,11 | 11,11 |
| **TH01** | 9.9,3 | 9.9,3 | 9.3,9.3 | 9.3,9.3 | 6,7 | 6,7 | 6,7 |
| **D13S317** | 10,12 | 10,12 | 11,12 | 11,12 | 12,12 | 12,12 | 12,12 |
| **D16S539** | 9,12 | 9,12 | 9,12 | 9,12 | 11,11 | 11,11 | 11,11 |
| **VWA** | 16,18 | 16,18 | 15,15 | 15,15 | 14,16 | 14,16 | 14,16 |
| **TPOX** | 11,12 | 11,12 | 9,11 | 9,11 | 9,9 | 9,9 | 9,9 |
| **AM** | X,X | X,X | X,X | X,X | X,Y | X,Y | X,Y |
| **DS5818** | 9,11 | 9,11 | 11,12 | 11,12 | 11,13 | 11,13 | 13,13 |
| **% identity**  (cell line vs tumor tissue) | **100** | | **100** | | **100** | | |
