# Supplementary material for: New Chondrosarcoma Cell Lines with Preserved Stem Cell Properties to Study the Genomic Drift During In Vitro/In Vivo Growth
Source: J Clin Med. 2019 Apr 4;8(4):455. doi: 10.3390/jcm8040455 (PMC6518242; doi:10.3390/jcm8040455)
Supplement: Supplementary file 1 [file jcm-08-00455-s001.zip › Rey et al - Table S3.docx]

| **Table S3. Overview of currently reported chondrosarcoma cell lines** | | | | | | | | | | | | |
| --- | --- | --- | --- | --- | --- | --- | --- | --- | --- | --- | --- | --- |
| **Cell line** | **Gender** | **Age** | **Sub-type (history)** | **Tumor location** | **Grade** | **passages** | **Tumorigenicity** | **IDH1** | **IDH2** | **P53** | **CDKN2A** | **Ref** |
| SW-1353 | F | 72 | central | humerus | II | >70 | yes | wt | R172S | V203L | del | ATCC® HTB94^TM^ |
| JJ012 | M | 39 | central | N/A | II | N/A | N/A | R132G | wt | G199V | del | 21 |
| ch-3573 | M | 25 | central | pelvis | II | >60 | yes | wt | wt | L201CfsX45 | N/A | 18 |
| OUMS27 | M | 65 | central | humerus | III | >70 | yes | wt | wt | mutated | del | 25 |
| ch-2879 | F | 35 | central | rib | III | >70 | yes | wt | wt | wt | del | 20 |
| BCSCH34 | M | 73 | central | tibia | III | >20.5 | no | R132C | wt | wt | del | 26 |
| BCSCH56 | F | 64 | central | humerus | III | >40 | yes | wt | R172S | wt | del | 26 |
| C3842 | M | 38 | Secondary (OD) | tibia | II | >30 | N/A | wt | wt | N/A | N/A | 22 |
| L835 | M | 54 | Secondary (OD) | radius | III | 50 | no | R132C | wt | wt | del | 27 |
| CDS06 | F | 61 | Secondary (OC) | pelvis | II | 20 | N/A | wt | wt | P72R | wt | This study |
| CDS11 | M | 61 | Secondary (OD) | scapula | I | 25 | yes | R132L | wt | P72R | del | This study |
| CAL78 | M | 76 | dediff | thigh | High | >70 | yes | wt | wt | E285_E286del | N/A | 19 |
| NDCS-1 | M | 38 | dediff | femur | High | N/A | yes | wt | wt | C242S | del | 24 |
| L2975 | M | 57 | dediff | femur | High | 60 | yes | wt | R172W | wt | del | 27 |
| L3252 | F | 52 | dediff | costa | High | 30 | no | wt | wt | wt | del | 27 |
| BCSCH03 | F | 74 | dediff | femur | High | >70 | no | wt | wt | Y107TfsX15 | del | 26 |
| CDS17 | M | 49 | dediff | hemipelvis | III | >35 | yes | wt | R172G | P72R; S215R | wt | This study |
| T-CDS17 | M | 49 | dediff | CDS17- xenograft line | III | >35 | yes | wt | R172G | P72R; S215R | wt | This study |
| OC: osteochondroma. OD: Ollier disease. N.A.: not available data. Dediff: dedifferentiated. Del: deleted | | | | | | | | | | | | |
