# Supplementary figures and images for: New Chondrosarcoma Cell Lines with Preserved Stem Cell Properties to Study the Genomic Drift During In Vitro/In Vivo Growth
Source: J Clin Med. 2019 Apr 4;8(4):455. doi: 10.3390/jcm8040455 (PMC6518242; doi:10.3390/jcm8040455)

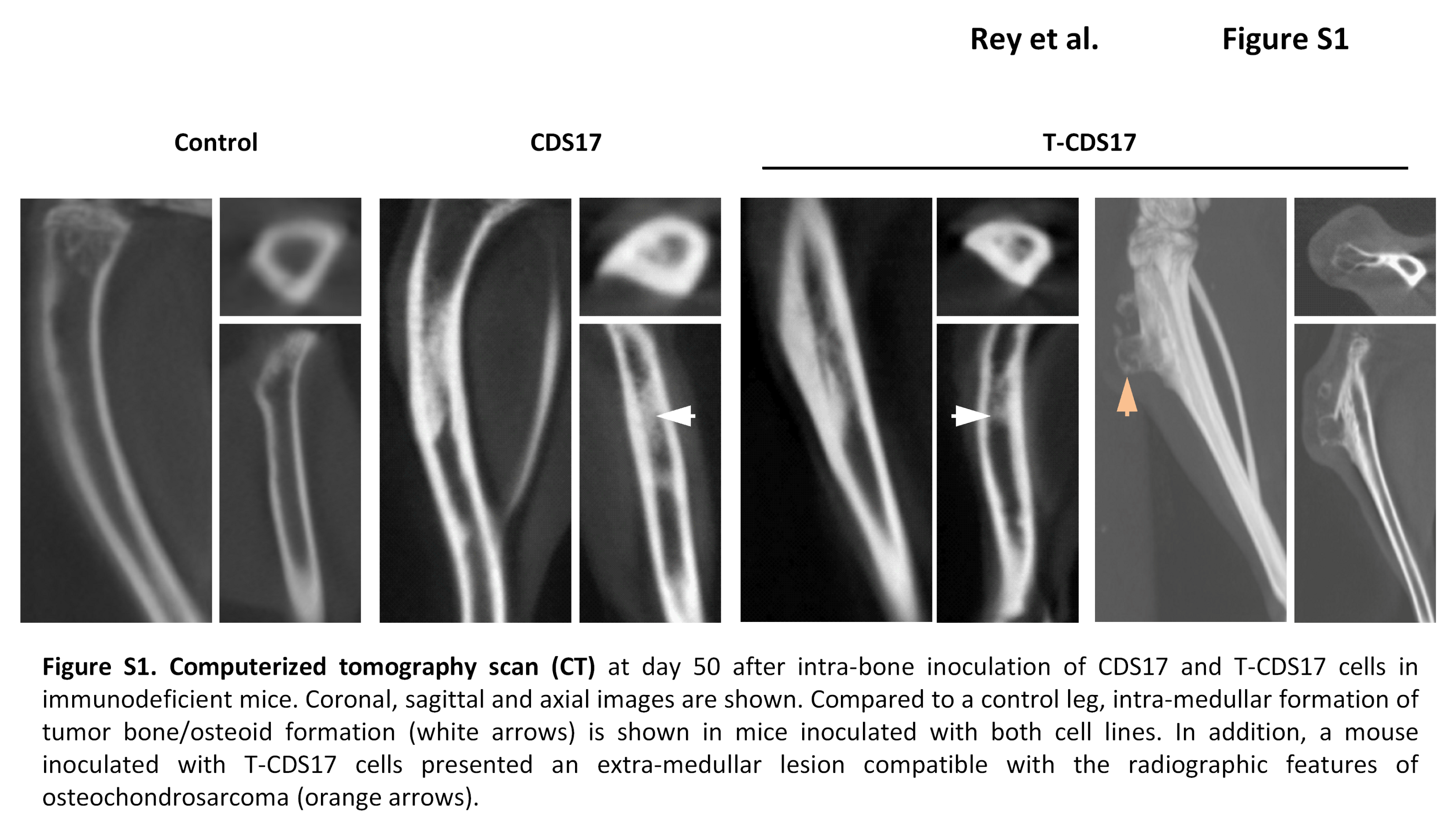

Supplement: Supplementary file 1 [file jcm-08-00455-s001.zip › Rey et al - Figure S1.tif]

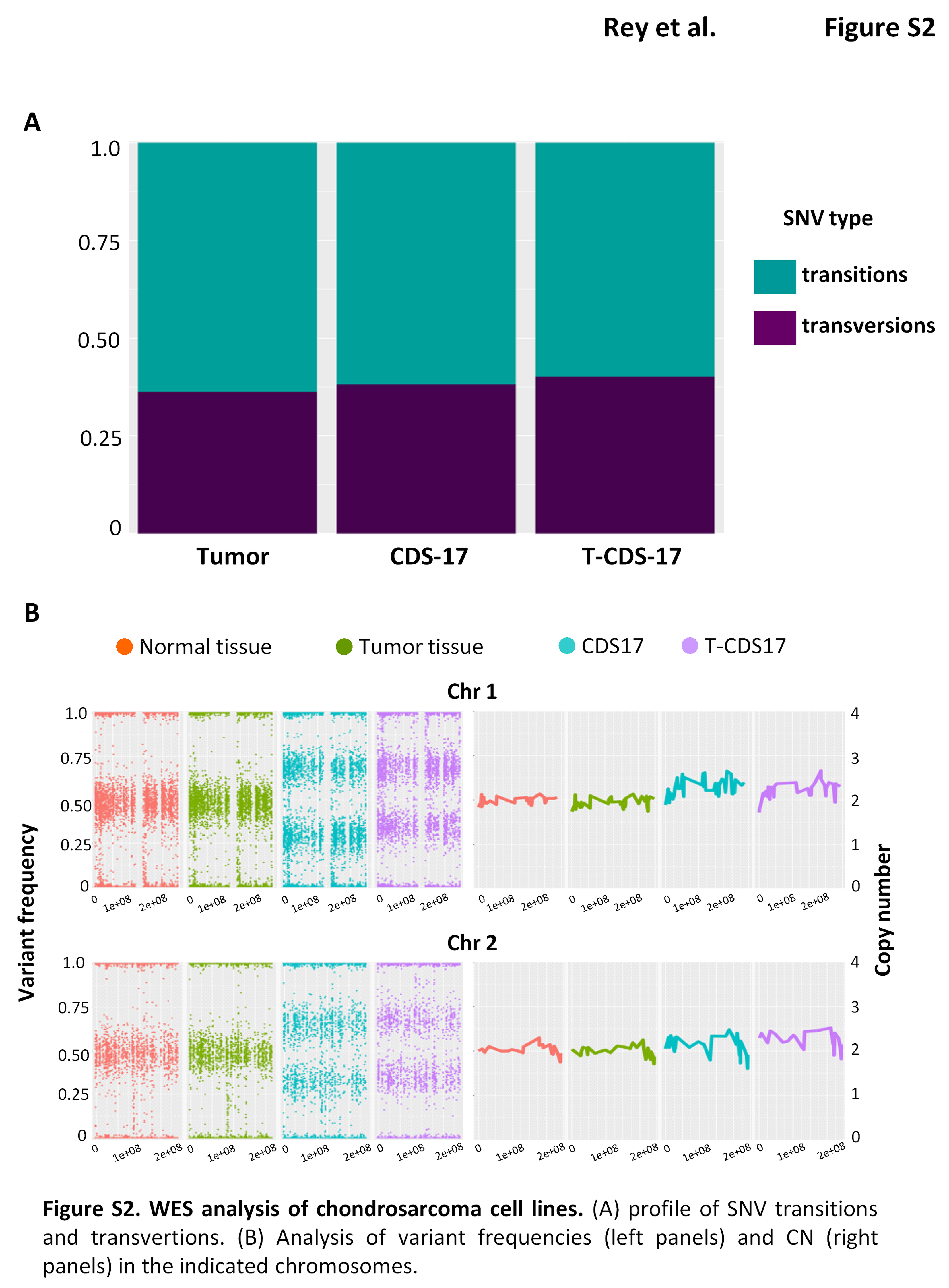

Supplement: Supplementary file 1 [file jcm-08-00455-s001.zip › Rey et al - Figure S2.tif]
